# Editorial for Special Issue “Antibacterial Resistance and Novel Strategies to Eradicate Bacterial Biofilms”

**DOI:** 10.3390/antibiotics11091184

**Published:** 2022-09-01

**Authors:** Theerthankar Das

**Affiliations:** Infection, Immunity and Inflammation Theme, Sydney Institute for Infectious Diseases, Charles Perkins Centre, School of Medical Sciences, The University of Sydney, Sydney, NSW 2006, Australia; das.ashishkumar@sydney.edu.au

Bacterial resistance to antimicrobial agents, including antibiotics, disinfectants, and detergents, is on the rise, with consequences associated with morbidity, mortality, and economic loss in the healthcare sector. The major factors that trigger resistance in bacteria include activation of the quorum sensing (QS) system among the bacterial population. Through the QS system, the biosynthesis of extracellular biopolymers (EPS) leads to complex and integrated biofilm formation and the hindrance of antibacterial penetration into the deep layer of biofilms. Other mechanism includes the production of antibiotic cleaving enzymes—such as β-lactamase and macrolide esterases, increase in the expression of efflux pump proteins that pump antibiotics out of the bacterial cell, and genetic changes that leads to alteration of antibiotic binding sites in bacteria, among other factors contribute to the antimicrobial resistance and the prevalence of infections. Understanding the resistance mechanism in bacteria is crucial, permitting the development of novel and innovative strategies for eradicating biofilms and controlling infections.

This Special Issue includes original research articles and reviews on challenging new molecules with antibacterial potential, which address antimicrobial resistance in bacterial biofilms.

The first section exhibits the potential of new antibacterial agents. Monteiro et al. (2022) investigated the impact of a laboratory-synthesized copper (II) complex (DRI-12) in combination with different antibiotics against the multi-drug-resistant strains of the food-borne pathogen *Salmonella Typhimurium* [1]. Their study showed that Cu(4-FH)(phen)(ClO_4_)^2^, (a selective copper (II) complex containing 4-fluorophenoxyacetic acid hydrazide and 1,10-phenanthroline) had improved synergism when combined with the antibiotics colistin (COL) and ceftriaxone (CFT), and when combined with COL/Aminopenicillins (AMP) in both the planktonic and the biofilm stages of bacteria. In the absence of DRI-12, different combinations of antibiotics—such as AMP/(sulfonamide) SUL/TET and AMP/ SUL/(tetracycline) TET/COL treatments—showed significantly less impact in comparison with DRI-12/antibiotics combinations [1].

A study by Jin and Yang (2022) presented the impact of the probiotic bacterium *Lactobacillus salivarius* LN12 cell-free supernatant (CFS) in combination with the antibiotics amoxicillin and clarithromycin on the biofilms and transcriptome of the gastrointestinal pathogen *Helicobacter pylori* [2]. The outcome of their study indicates that when the CFS of *L. salivarius* is combined with amoxicillin/clarithromycin, the overall destructive effect of antibiotics increases significantly (in comparison to the impacts of amoxicillin/clarithromycin by themselves); these improvements included a decrease in biofilm biomass, a threefold reduction in CFU/ml, and changes in (4-day-old) *H. pylori* biofilm morphology. Most interestingly, a transcriptome analysis of *H. pylori* biofilms using RNA-Seq and RT-qPCR techniques indicated that, when high-bacterial-density CFS is combined with antibiotics, the pathogen produces a more robust oxidative stress response by increasing NADH-related genes and downregulating its flagellar-assembly-related and QS-related receptor genes. In conclusion, the authors stated that a higher dose of probiotics above the “threshold” is essential to see the evident positive impact in reducing *H. pylori* pathogenicity [2].

In vitro experiments conducted by Manoharan et al. (2021) demonstrated that N-acetyl cysteine (NAC) is an excellent antibacterial agent that can inhibit the invasion of uropathogenic *Escherichia coli* into the epithelial cells of the human bladder [3]. The intrinsic acidity of NAC induced damage in the bacterial (*E. coli* and *Enterococcus faecalis*) cell membrane, thereby promoting cell lysis and inhibiting biofilm formation. Simultaneously, NAC was shown to enhance antibiotic (ciprofloxacin) efficacy in reducing bacterial viability and growth. Interestingly, NAC exhibited no cytotoxic impact on the epithelial cells of the bladder even after continuous exposure for 48 hours. Their study concluded that NAC could potentially prevent the formation of intracellular bacterial/biofilm communities inside bladder epithelial cells and promote efficacious treatment for urinary tract infections against bacterial pathogens [3].

Novel nitro-heteroaromatic compounds synthesized by Koenig et al. (2021) showed enhanced antimicrobial activity against *Staphylococcus epidermidis* and *Pseudomonas aeruginosa* biofilms [4]. This study modified the structures of commercial antimicrobials Nitazoxanide, nitrofurantoin, and furazolidone. Out of approximately 20 analogues in this study, the most critical impact was seen with analogue 1b (2-Acetoxy-5-chloro-N-(5-nitrothiazol-2-yl) benzamide), which has a chlorine group substituent at the 5-position of the Nitazoxanide ring. Additionally, with analogue 7b (2-Amido-5-chloro-N-(5-nitrothizol-2-yl) benzamide), the 2-acetoxy substituent in 1b was replaced with the primary amine group (analog 7b). Both these analogues (1b and 7b) boosted antibacterial activity in the planktonic and biofilm stages [4].

The second section of this Special Issue comprises review articles. The article by Cuenca et al. (2021) focused specifically on functional bacterial amyloids, which were found to be an essential part of the biofilm matrix [5]. The highlight of this review is the finding that amyloid fibrillar proteins with a β-sheet secondary structure can be considered as structural scaffolds that provide viscoelasticity to the biofilm matrix. Amyloids are classified into intrinsic and facultative amyloids; one example is Curli (CsgA and CsgB subunits that structurally form amyloids), which is encoded by operons *csgBAC* and *csgDEFG*. To inhibit amyloid functions and disrupt the biofilm matrix, several anti-amyloid compounds (e.g., polyphenols, curlicides) that are involved in the inhibition of amyloid production or interfere with their assembly have been developed [5]. 

In another review, Abdulkareem et al. (2022) compared the efficacy of classic biofilm removal vs. novel or alternative strategies for their removal in periodontal disease [6]. The authors compared many clinical case studies and showed the efficacies and challenges in treating biofilm-associated periodontal disease. The dental biofilm plaque is considered to be the primary factor in periodontal disease. The gold-standard method for removing dental plaque involved the use of mechanical periodontal/subgingival debridement. The main objective of this therapy is to remove the bulk of the dental biofilm, together with calculus, to reduce inflammation. To augment the impact of periodontal debridement, adjuvant treatment, including standard antibiotics, has frequently been utilized. However, the clinical outcomes are under question in the light of some case studies which have shown no additional improvements in using antibiotics together with the mechanical debridement technique. It is predicted that the main factor for antibiotics’ failure is bacterial resistance to antibiotics [6].

This Special Issue presents articles—written by scientists with multidisciplinary backgrounds—which are focused on bacterial resistance to antimicrobial challenges. These papers assert the need to be innovative in tackling the rising problems of antimicrobial resistance and superbug development. The content in this Special Issue will serve as an excellent reference for researchers, scientists, and doctors in the fields of microbiology and bacterial infectious diseases.
